# Categorizing and comparing psychophysical detection strategies based on biomechanical responses to short postural perturbations

**DOI:** 10.1186/1475-925X-9-58

**Published:** 2010-10-08

**Authors:** Viprali V Bhatkar, Joseph D Skufca, Rakesh B Pilkar, Christopher M Storey, Charles J Robinson

**Affiliations:** 1 Clarkson University, Potsdam, NY, USA; 2 Louisiana State University School of Medicine, Shreveport, LA, USA; 3 Syracuse VA Medical Center, Syracuse, NY, USA

## Abstract

**Background:**

A fundamental unsolved problem in psychophysical detection experiments is in discriminating guesses from the correct responses. This paper proposes a coherent solution to this problem by presenting a novel classification method that compares biomechanical and psychological responses.

**Methods:**

Subjects (13) stood on a platform that was translated anteriorly 16 mm to find psychophysical detection thresholds through a Adaptive 2-Alternative-Forced-Choice (2AFC) task repeated over 30 separate sequential trials. Anterior-posterior center-of-pressure (APCoP) changes (i.e., the biomechanical response R_B_) were analyzed to determine whether sufficient biomechanical information was available to support a subject's psychophysical selection (R_Ψ_) of interval 1 or 2 as the stimulus interval. A time-series-bitmap approach was used to identify anomalies in interval 1 (a_1_) and interval 2 (a_2_) that were present in the resultant APCoP signal. If a_1 _> a_2 _then R_B _= Interval 1. If a_1 _< a_2_, then R_B_= Interval 2. If a_2 _- a_1 _< 0.1, R_B _was set to 0 (no significant difference present in the anomaly scores of interval 1 and 2).

**Results:**

By considering both biomechanical (R_B_) and psychophysical (R_Ψ_) responses, each trial run could be classified as a: 1) HIT (and True Negative), if R_B _and R_Ψ _both matched the stimulus interval (SI); 2) MISS, if R_B _matched SI but the subject's reported response did not; 3) PSUEDO HIT, if the subject signalled the correct SI, but R_B _was linked to the non-SI; 4) FALSE POSITIVE, if R_B _= R_Ψ_, and both associated to non-SI; and 5) GUESS, if R_B _= 0, if insufficient APCoP differences existed to distinguish SI. Ensemble averaging the data for each of the above categories amplified the anomalous behavior of the APCoP response.

**Conclusions:**

The major contributions of this novel classification scheme were to define and verify by logistic models a 'GUESS' category in these psychophysical threshold detection experiments, and to add an additional descriptor, "PSEUDO HIT". This improved classification methodology potentially could be applied to psychophysical detection experiments of other sensory modalities.

## Background

A major goal of the psychophysical experiments that we carry out in our laboratory has been to find if changes in one or more biomechanical or physiological variables correlate with the ability to correctly detect small anterior translational perturbations (≤16 mm) of the platform upon which a subject was standing.

The primary test protocol was based on a 2-Alternative-Forced-Choice (2AFC) procedure, where the subject was forced to choose which of two sequential intervals exclusively contained the platform perturbation. The displacement length was fixed, and the platform acceleration was iterated over a sequence of tests to identify a perceptual threshold. This iteration was carried out via a modified Parameter Estimation Sequential Testing protocol (PEST) [1]. However, a major difficulty existed in determining whether a correct response reported by the subject indicated an underlying ability to detect a perturbation or indicated a guess, especially when an acceleration level was close to detection threshold. Hence, we sought profiles in our data that could be used to distinguish biomechanical changes seen during "real" psychophysical detection of the stimulus from those "correct" psychophysical responses caused by other conditions, including chance guesses. We made an explicit assumption in this paper that a stimulus that was perceived must have had a concomitant physiological, biomechanical or neurophysiological response. The logic of this assumption is as follows:

IF a stimulus evoked a certain biomechanical response at some threshold, P_B_, AND IF there existed a psychophysical detection threshold, P_Ψ_, for that stimulus, AND IF detection was somehow linked to that biomechanical response,

THEN studying the biomechanical and psychophysical responses together would enable us to account for guesses and other non-congruent responses.

This rule might be equivalent to looking a pupillary response to light flashes of varying intensities, and tying those responses to psychophysical detectability. As noted in the rule, we employed two different signal detection mechanisms. First was a psychophysical one where a subject responded by choice of interval where he/she thought that they felt the move. The second mechanism was in terms of a biomechanical response, the Anterior-Posterior Center of pressure (APCoP). We did not claim that the biomechanical response that we chose (i.e., APCoP) was the sole response that could occur, or that detection depended on its occurrence. But we started with that premise to develop our theory and methods. In later papers, we will look at other variables and across multiple variables.

The time-series trajectories of the Center of Pressure (CoP) (related to the vertical projection of the Center of Mass onto the surface upon which this person stands) have been analyzed by many as a measure of postural stability [2-5]. Nearly every modern study on postural control has collected such CoP data as crucial to the experimental analysis. When studying how the body reacts to large perturbations of the platform, the CoP signal provided a clear indication of postural control response that can be directly correlated with the stimulus. However, under quiet standing conditions (with no platform motion as stimulus), the CoP signal still showed significant transient behavior, and could be modeled as a random walk [6]. Research described in this paper focused on the intermediate situation where the body was subjected to small perturbations (meant to mimic the start of a slip or stumble) in an attempt to understand postural control at the borderline of movement perception. Our previous studies have primarily addressed the identification of a perception threshold - the minimum platform motion that could be detected by a test subject [7-10].

A fundamental unsolved problem in psychophysical detection experiments is in discriminating guesses from the correct responses. This paper proposes a coherent solution to this problem by presenting a novel classification method that compares biomechanical and psychological responses. This present paper focuses on newly developed analysis techniques that allow us to characterize and classify APCoP behavior in response to small perturbations and to improve our understanding of the relationship between the APCoP signal and perception threshold.

## Methods

In these methods, we outline a new probability model for 2AFC experiments that captures our refined categorization, and give a detailed description of how we identify anomalous behavior. For analysis, our specific computational technique required significant data dimensionality reduction, achieved by symbolic representation of abstracted data. Our other innovation (presented here) is a modified time-series-bitmap (TSB) approach [11] to identify anomalies present in the biomechanical response (i.e., a change in APCoP). It identified "anomaly" not with respect to the full data set, but rather, with respect to a small moving window, providing an estimate of the instantaneous information content, which we could then use to evaluate whether there was sufficient information to differentiate between the two stimulus presentation intervals. Traditional low pass filtering technique could not be used as it does not really amplify the biomechanical response behavior, resulting into failure of differentiating responses. Original work of TSB method was modified by separately the anomaly score into the two separate anomaly score measures.

### Subjects

We analyzed the performance of 13 healthy adults [2 M, 11 F] over 49 y.o. [median age 58 y.o., (min/max 50/67)] and without diabetes or lower limb peripheral neuropathy (as verified by clinical nerve conduction velocity testing). This data was collected at the Shreveport, Louisiana, VA Medical Center under an Institutional Review Board (IRB) approved protocol. Subjects were screened for balance, sole tactile acuity, clinically determined nerve conduction velocities, and other measures as outlined in [12,13]. All screens were within normal limits.

### Experimental Procedures

To avoid extraneous clues due to movement vibration for these small perturbation experiments, we developed test hardware and software that we collectively call SLIP-FALLS-STEPm (for Sliding Linear Investigative Platform For Assessing Lower Limb Stability with Synced Tracking, EMG and Pressure measurements). This equipment performs precisely controlled vibration-free horizontal translations through the use of air bearings and a linear motor [14]. Processed SLIP-FALLS-STEPm data from past experiments provided various time-series signals that include position, acceleration, APCoP, and EMG data. For each subject, a maximum of 30 trials are performed per run to prevent fatigue, and each trial collects these and other time-series data.

**Figure 1:**
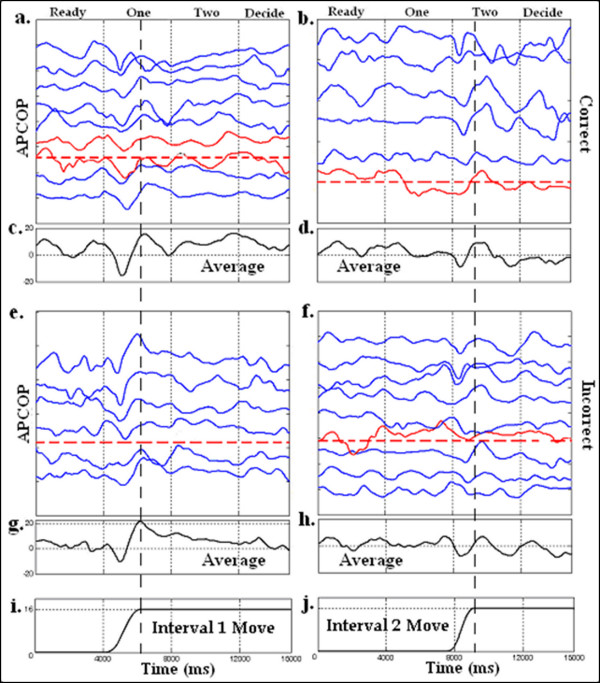
**APCoP (mm) plots from a 30 trial run for subject f50z031**. Categorization of trials into CORRECT (a, b) and INCORRECT (e, f) for Interval 1 (a, e) and Interval 2 (b, f). Plots (c, d, g, h) give the point-by-point ensemble average. The scale of (c, d, g, h) also applies to the individual plots in the raster. The individual time series in each raster are sorted such that those with the highest acceleration value are at the top of each plot. The platform position (mm) signals from the highest and lowest acceleration values tested are shown in i and j. The horizontal dashed lines illustrate the experimentally determined detection threshold, while the vertical dashed lines are set at the end of the averaged position signal in Interval 1 and 2. Observe that the ensemble average of CORRECT versus INCORRECT runs shows very similar APCoP response to the stimulus, indicating that classifying based on correctness of response may be of limited value for data mining of the average signal.

We determined psychophysical threshold by using a 2-Alternative-Forced-Choice (2AFC) protocol in which the subjects were required (i.e., "forced") to choose in which one of two sequential intervals that they perceived the presented perturbation. In this protocol, the subject sequentially received the commands "Ready", "One", "Two," and "Decide", at the start of intervals of 3 to 6 s each, with a stimulus either in interval One or Two [15-18]. After the word "Decide", the subject pressed a telemetered doorbell button once or twice to signal in which interval he/she felt that the stimulus occurred. The length of the move was 16 mm, and a set of maximum 30 trials was collected for each subject. Data collected included various signals like platform position, and acceleration, a subject's APCoP and medial-lateral CoP (MLCoP), as well as horizontal ground reaction forces and tri-axial head acceleration. However, the variable of interest for the analysis presented in this paper was APCoP. Data were sampled at 1000Hz and converted offline into engineering units, filtered and downsampled to 100 Hz. A typical anterior horizontal 16 mm position move time-series profile is shown in Figure 1 (i, j), for which the acceleration profile resembles a single sinusoid [14]. The test stimulus variable (i.e., the Peak Acceleration value of the positive [or negative] half sinusoid-like profile) was iterated to threshold using a modified PEST routine [1].

### Modeling 2AFC Behavior

In our 2AFC experiment, a subject was presented with two sequential intervals, one of which contained a stimulus and the other of which did not. The subject was required to select an interval, and the experimental outcome recorded as to whether the choice was correct or not. Note that the designation of "correct" simply implied that the stimulus interval was correctly signalled. It did not imply that the subject actually detected the stimulus. When used to evaluate the subject's ability to detect some stimulus (threshold experiments), the standard probabilistic model for "correct" response is

P(correct)=P(correct|non-detect) P(non-detect) +P(correct|detect) P(detect)

The assumption was that if the stimulus was not psychophysically detected, then the probability of choosing the correct interval was p = 1/2 (assuming an unbiased experiment). Conversely, if the signal was detected, then the subject would have answered correctly. Letting z indicate the probability of detecting the signal yielded the simplified model

(1) P(correct)=(1−z)/2+z=(1+z)/2

The immediate result of this model was that any particular correct answer could not be viewed as "detection," because half of the time the subject would have been guessing correctly, even when the stimulus was not observable. This is why classical detection thresholds were set at 75% on better. The procedures for implementing a 2AFC test with a threshold determination scheme (PEST) explicitly accounted for this model.

For our SLIP-FALLS data analysis, our goal was to data-mine the detailed time series to better understand the body response to low-level perturbations. Because the signal-to-noise ratio was quite small, we would have been working with a significant stochastic component in the measured APCoP response [6]. This, in turn, meant that ensemble averaging would have been appropriate. However, simply averaging across "correct" or "incorrect" responses failed to adequately resolve the difference between guessing (correctly or incorrectly) and correctly responding to a stimulus signal. Figure 1 shows the APCoP data for a 50 years old female subject, clustered by stimulus interval, and by whether the subject's response was correct or incorrect. Trials were sorted by the magnitude of the stimulus acceleration. By visual inspection, it appeared that many of the "correct" response runs had less signal strength than some "incorrect" runs, with the ensemble averages (Figures 1c,d,g,h) showing very little difference between the two cases.

We propose here an alternative probabilistic model based on the following partition of observation space into three distinct categories: (a) If the stimulus was very small, the signal could also have been so much smaller than noise such that there was no apparent stimulus signal present in the APCoP data, indicating that the subject made a pure guess (denoted **G**), (b) The stimulus resulted in sufficient information in the APCoP data that the stimulus was *detectable*, denoted **D**. (Note - *detectable *indicated the presence of signal in the APCoP data that was above some noise threshold, not that the subject perceived that signal.); (c) The APCoP signal could have been above threshold; but not due to the stimulus signal, such that the APCoP signal was *misleading *(denoted **M**), in that it indicated a signal that caused the subject to choose contrary to the actual stimulus. [Note: In some sense, this categorization lacks precision because it does not specify how much signal was required in the APCoP signal to assign the designation "detectable." In the subsequent section, we will make those definitions precise, basing that classification on the specific measurement of our anomaly detection algorithm.]

If we denote a "correct" response by **C**, we have new probability model

(2) $$P(C)=\underset{\text{Guess}}{\underset{︸}{P(C|G).P(G)}}+\underset{\text{Hit}}{\underset{︸}{P(C|D).P(D)}}+\underset{\text{Pseudo}\;\text{Hit}}{\underset{︸}{P(C|M).P(M)}}$$

where again we have assumed an unbiased experiment with respect to guessing. Each of these summands now associates to a specific classification of a particular 2AFC experimental run: (a) a correct "guess," (b) a "hit," and (c) a "pseudo-hit" (defined later), as labeled above. The alternative outcomes from the experiments occurred when the subject chose the incorrect outcome for the stimulus, denoted as **C**', modeled as,

(3) $$P(C')=\underset{\text{Guess}}{\underset{︸}{P(C'|G).P(G)}}+\underset{\text{Miss}}{\underset{︸}{P(C'|D).P(D)}}+\underset{\text{False}\;\text{Positive}}{\underset{︸}{P(C'|M).P(M)}}$$

with summand descriptors of (a) incorrect "guess," (b) a "miss" (because the subject *missed *the detectable signal), and (c) "false positive," (where the subject responded consistent with the misleading CoP signal).

### Time Series Bitmap Based Analysis

#### Background

Our hypothesis was that an unexpected or anomalous pattern in a subject's APCoP time-series data might have been used by a subject to quantify a stimulus, shedding light on detection strategies behind a given subject's psychophysical response. In this section, we provide an explicit description of our processing techniques that support this hypothesis. Our basic approach remained the same as the preliminary report of Bhatkar et al. [19], but we have fine-tuned the parameters of the algorithm to increase the effectiveness of the method as a classifier of the APCoP.

#### Preprocessing, Dimensionality Reduction, and Data Abstraction

To reduce the time-series from a large *n *dimension to a much smaller *w *dimension, the data was divided into *w *equal sized "frames". A Piecewise Aggregate Approximation (PAA) was used to abstract the data, where a time-series C of length n is represented in a *w*-dimensional space by a vector$\bar{C}=\bar{c_{1}},...,{\bar{c}}_{w}$. The i^th ^element of $\bar{C}$ was calculated by the Equation 4 [20]:

(4) $$\bar{c_{i}}=\frac{w}{n}\sum_{j=\frac{n}{w}(i-1)+1}^{\frac{n}{w}-i}c_{j}$$

**Figure 2:**
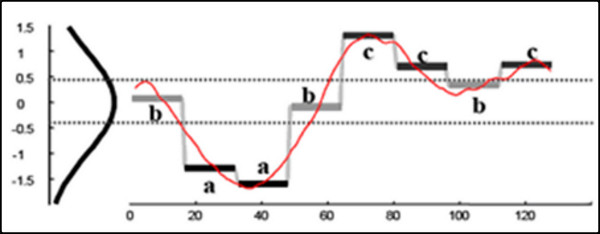
**A real valued time-series can be converted to the SAX word baabccbc**. The y-axis represents z-scores. *Note*. Adapted with permission from "Assumption-Free Anomaly Detection in Time Series," by Wei et al. [11].

PAA replaced equally sized frames by their mean values, discretized in the time domain. After applying the SAX algorithm, the PAA data was then spatially scaled by z-score, where each spatial interval was represented by a letter from a finite alphabet, producing a SAX word representation of the data stream. The alphabet size, σ, could be chosen arbitrarily. For σ = 3, the alphabet would be taken as {a, b, c}. Figure 2 shows a short time-series being converted into SAX word **baabccbc **[11]. Because our data consisted of lengthy time-series, the ability to convert subsequences of time-series data into the much lesser dimensional SAX word representation facilitated anomaly detection.

#### Time Series Anomaly Detection

**Figure 3:**
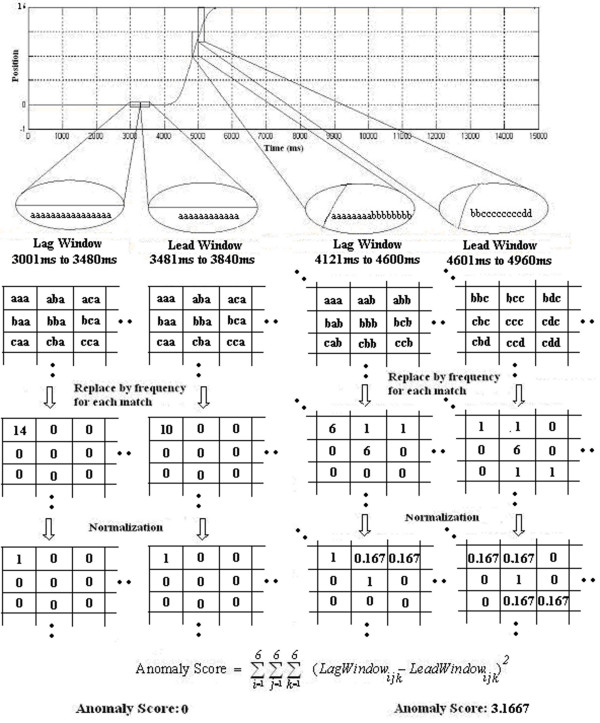
****Method illustrating Time-Series-Bitmap Algorithm****. Illustrating conversion of data subsequences into SAX symbols to calculate anomaly scores for each instance of the lag and the lead windows using the known time-series data of platform position taken from a move in Interval 1. *Note*. Adapted with permission from Bhatkar et al, 2006 [19].

Our approach to time-series anomaly detection was also inspired by work done by Li Wei et al. Two adjacent windows called the lead window and the lag window were slid across the time-series. Each window was converted to SAX representation as above, and frequencies of SAX subwords were calculated (see Figure 3). It was the matrix of frequency information that was viewed as the time-series-bitmap (TSB) for the specified window. The "distance" between two windows was computed as the Frobenius norm on the matrix of word frequencies, and that value was recorded as the *anomaly score *at that instant. As the windows moved along the time series, a new data point was ingressed and an old data point egressed, with the frequency matrices updated at each step, providing for efficient computation of the time-series scores [19]. We highlight that our technique and choice of window sizes was different from the approaches presented by Wei et al. and Bhatkar et al. By computing over the small lead and lag windows, our anomaly computation described a local rate of change of signal character, rather than measuring a difference from the normal behavior of the full sequence. Specific parameters used for analysis are listed in Table 1.

**Table 1:** The list of the parameters used for the modified TSB algorithm.

Parameter	Value
bin size	30

word length (lag)	16

lag window size	480 ms

word length (lead)	12

lead window size	360 ms

alphabet size	6 {a, b, c, d, e, f}

#### Algorithm Implementation

**Figure 4:**
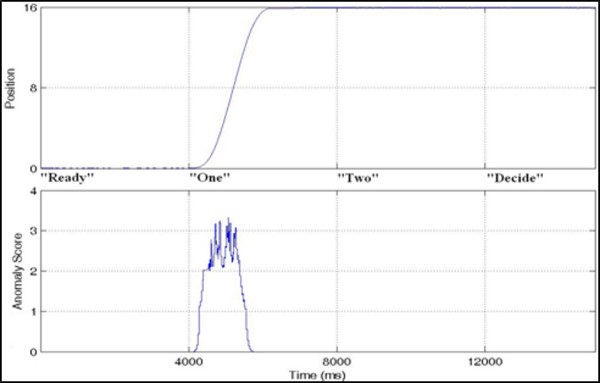
****Time series record of the platform position (a) and anomaly score (b)****. Both signals indicate where actual movement has occurred. The dotted lines in this and subsequent figures denote the four test intervals.

**Figure 5:**
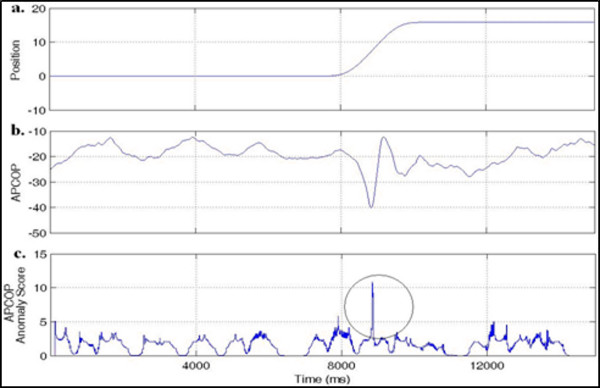
**(a) Platform movement, (b) associated APCoP signal, and (c) the computed anomaly score**.

The anomaly score could be associated to the relative entropy of the empirical distributions of the lead and lag windows, and could be thought of as a generalization of the derivative. When the signal was changing rapidly in character, one could expect a large anomaly score. Analysis of the anomaly score for PLATFORM POSITION is shown in Figure 4 to illustrate this ability to detect a change, albeit in platform position. Our goal was not only to find the APCoP changes that were influenced by platform perturbations, but also to relate these APCoP excursions to the correctness of the subject's decision for movement detection. Figure 5 is a typical plot of the change in platform position, the resultant change in APCoP, and the anomaly score for that APCoP. Note that although there was a clear deterministic response apparent in the APCoP for this run, such a clear deterministic response was not always present.

#### Use of anomaly score to classify biomechanical response

A subject's response to a perturbation could be characterized in two different ways. One is the *psychophysical response*, R_Ψ, _indicating in which interval the subject *perceived *that the movement occurred, experimentally collected by simply having the subject respond by button press. Another is the subject's *biomechanical response*, R_B_, which can be a neurophysiological (i.e., EMG) or a biomechanical (e.g., APCoP) response to a perturbation. We chose to use the biomechanical response to classify signals. We performed this classification by focusing on APCoP and its associated anomaly scores. We considered this aspect to be a crucial contribution of our work. We tailored the numerical classifier to act in accordance within the psychological rules imposed by the experiment, such that it modelled how the body might by using that data to perform the 2AFC task of the experiment.

In our classification system, a_1 _and a_2 _were the root mean square (RMS) values of the anomaly score during interval 1 and interval 2 respectively, R_B _was calculated based on the difference between these two values. When the difference between the two anomaly scores was greater than a specified threshold value (the value of *threshold *was set to be 0.1, but that choice is discussed below), the R_B _value was set to the stimulus interval with the greater anomaly score. If a_1 _> a_2, _then R_B _was set to 1. Conversely, if a_1 _< a_2_, then R_B _was set to 2. When this difference was less than threshold (i.e., there was an insignificant distinction between interval 1 and interval 2 anomaly scores), R_B _was set to 0.

**Figure 6:**
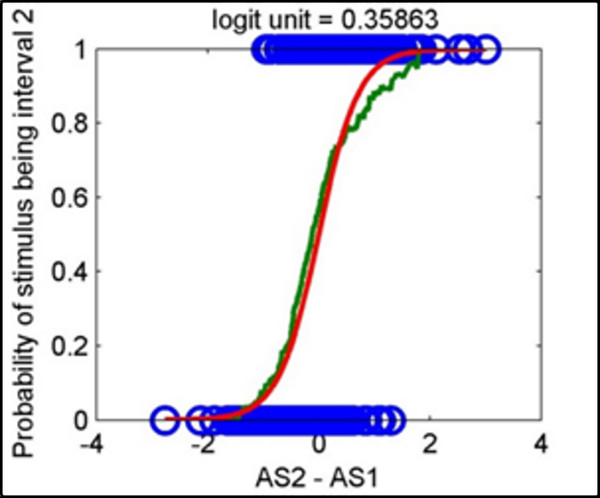
**The relationship between difference in anomaly score and stimulus interval**. Observed data from 13 subjects (circles), where each data point represents a single run that is plotted as either a 0 (if stimulus was in interval 1) or 1 (if stimulus in interval 2). The difference in the anomaly score (anomaly score 2 - anomaly score 1) forms the horizontal axis, The green curve shows a moving average of the data, representing an empirical likelihood that the anomaly score difference would indicate a interval 2 stimulus. The red curve shows a logistic model fit to that data.

Our justification for using an anomaly score to classify a biomechanical response was based on the strong correlation between the anomaly score difference and the actual stimulus interval. Figure 6 illustrates this relationship, where data from 372 runs across 13 subjects were analyzed for anomaly score differences. A positive difference was associated with increased APCoP anomaly in the second interval, and should have been correlated with the actual stimulus in that interval. An empirical curve (based on a moving average) indicated that the likelihood that the stimulus was in interval 2 increased monotonically in a sigmoidal fashion as the anomaly score difference increased. A logistic regression line to this data is also shown. The logistic intercept (equal probability of interval 1 or 2) regression coefficient of -0.02 was statistically indistinguishable from 0, supporting the conclusion that the experiment was unbiased between the two intervals. The anomaly score correctly indicated the stimulus interval at an accuracy rate of 81.4%, which is significantly better than the subject's response accuracy of 71.8%.

Using the logistic regression model of the data, we observed that if a_2 _- a_1 _< 0.1, the probability that stimulus was in interval 2 ranged from 43% to 57%. With so little discriminating power, we considered this to be equivalent to guessing, leading to our choice of assigning 0.1 as the threshold for R_B_. Restricting to cases that exceeded this threshold, the classification accuracy rose to over 83%. We could now rigorously define "detectable" as those cases where R_B _matched the stimulus and "misleading" when R_B _was counter to the stimulus, with "guess" defined by an anomaly score difference being below threshold.

## Results

### Classification of detection strategies using both biomechanical and psychophysical responses

**Figure 7:**
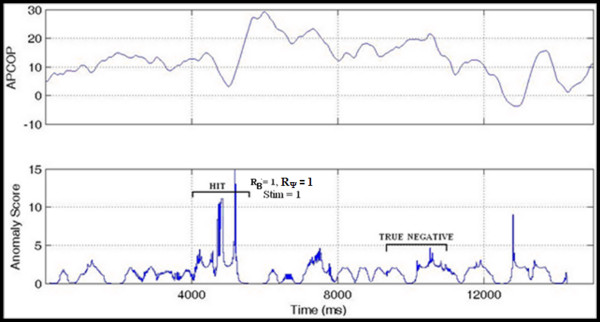
**Interval 1 HIT**. A high anomaly score is seen in interval 1 (the Stimulus Interval) indicating a HIT. Interval 2 shows a TRUE NEGATIVE with low anomaly (Non-stimulus Interval).

Our new categorization considered three factors: (a) the subject's psychophysical report of his/her detection of a stimulus interval; (b) the actual interval in which the stimulus occurred; and (c) the subject's biomechanical response, based on the differentiability of information content of the two presentation intervals, regardless of whether that signal was due to a stimulus presentation or noise. Our categorization terminology is defined below, expressed in terms of our specific platform perturbation experiment and collected CoP data:

'HIT': Where the subject's psychophysical response matched with both the biomechanical response and the actual movement interval. If R_B _and R_Ψ _both match to the stimulus interval, we assume that the subject has responded correctly to actual movement of the body in response to the stimulus (Figure 7).

'TRUE NEGATIVE': Congruent with a HIT above, where the subject did not choose the non-stimulus interval. The biomechanical response in that stimulus was far below that of the stimulus interval. If R_B _and R_Ψ _both matched to the stimulus interval, then non-stimulus interval is considered as true negative (Figure 7).

**Figure 8:**
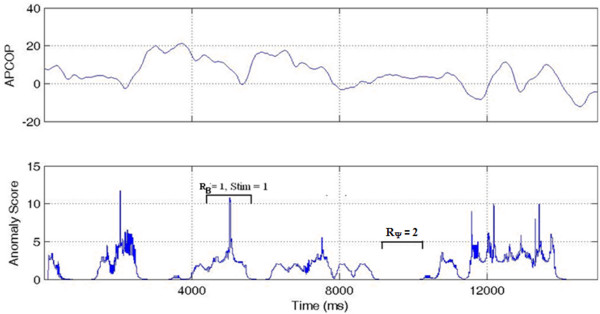
**Interval 1 MISS**. A MISS is indicated since a high anomaly score was seen in interval 1 (the Stimulus Interval) but subject selected Interval 2.

'MISS': Where the biomechanical response matched the movement interval, but the subject's psychophysical response was incorrect, indicating that the subject might have missed the potential biomechanical indicators. R_B _matched the stimulus (indicating that sufficient signal was present for detection) but the subject's reported response was incorrect (Figure 8).

**Figure 9:**
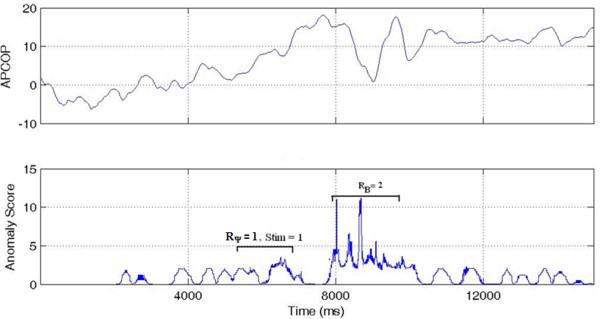
**Interval 1 PSEUDO HIT**. While the stimulus was in Interval 1 and the subject chose that interval, the higher anomaly score was in interval 2. This was thus scored as a Interval 1 PSEUDO HIT.

'PSEUDO HIT': Where the subject psychophysically selected the stimulus interval, but the biomechanical response (anomaly) was present in non-stimulus interval. If the subject reported the correct stimulus interval, but R_B _was observed in the non-stimulus interval, this indicated that subject's decision might have been affected by something other than body movement measured by CoP changes (Figure 9).

**Figure 10:**
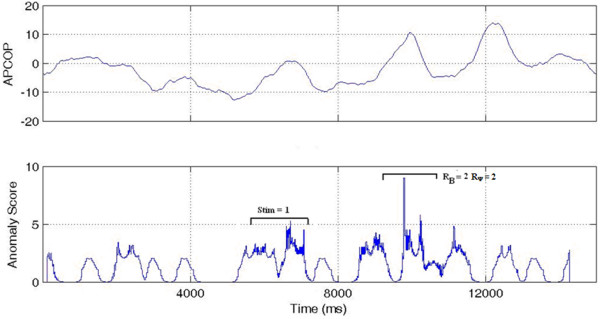
**Interval 1 FALSE POSITIVE**. With the stimulus in Interval 1, the higher anomaly score was in the interval 2 and the subject also selected Interval 2. This was thus coded as an Interval 1 FALSE POSITIVE.

'FALSE POSITIVE': Where the subject's psychophysical response matched the biomechanical response, but where both indicators were contrary to the actual stimulus interval. We interpreted this case as that the subject responded to a "noisy" biomechanical signal, where the noise caused a spike above detection threshold. In these trials, R_B _= R_Ψ_, but both were associated to the non-stimulus interval. We interpreted these trials as the subject responding to actual body motion, but with that motion not due to the stimulus (Figure 10).

**Figure 11:**
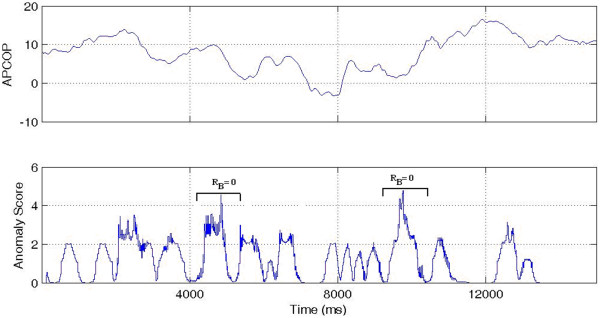
**GUESS**. High APCoP anomalies existed in both the intervals, with correct detection of the stimulus interval.

'GUESS': Where no significant difference existed in the biomechanical signal anomalies between stimulus and non-stimulus intervals, such that the APCoP provided ambiguous information to the subject. If no other information were to be available to the subject, then regardless of whether their psychophysical response was correct or not, it could be viewed as a chance result (Figure 11).

**Figure 12:**
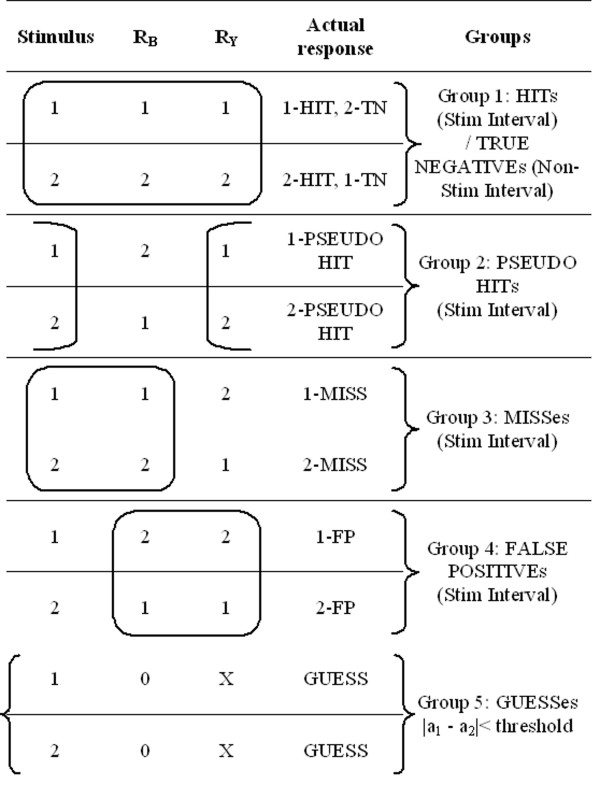
**Categorization of trials based on Stimulus Interval**.

We employed this classification scheme to perform ensemble averaging across multiple trials and to find that it appeared to provide a robust method for grouping behavioural response. By considering both the biomechanical and psychophysical responses, we were able to classify each trial run at the resolution indicated by Equation 2 and Equation 3. Though only six categories were mentioned above, there were actually eight combinations present together for both the intervals. Figure 12 summarizes this categorization for a standard 2AFC test.

**Figure 13:**
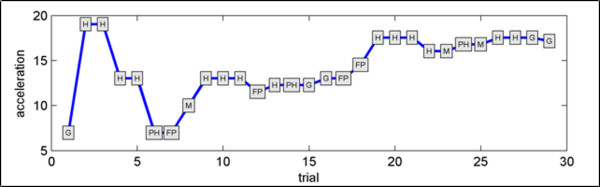
**PEST iterations for 66 year-old female subject f66z067 (refer to Table 2)**.

Table 2 implements this classification for a 66 year old female [f66z067] for a set of 29 trials. Figure 13 shows this subject's PEST iteration process towards psychophysical threshold.

**Table 2:** Categorization of 30 trials of a 16 mm perturbation.

Trial	**Accel**.	Stim	R_B_	R_Y_	CPh	A_Score Int 1	A_score Int 2
5	13.02	1	1	1	1-HIT, 2-TN	1.94	1.51

9	13.02	1	1	1	1-HIT, 2-TN	2.17	1.81

10	13.02	1	1	1	1-HIT, 2-TN	2.10	1.89

11	13.02	1	1	1	1-HIT, 2-TN	3.38	1.87

20	17.54	1	1	1	1-HIT, 2-TN	1.91	1.08

26	17.54	1	1	1	1-HIT, 2-TN	1.98	1.46

2	19.04	2	2	2	2-HIT, 1-TN	1.45	3.18

3	19.04	2	2	2	2-HIT, 1-TN	1.95	2.56

4	13.02	2	2	2	2-HIT, 1-TN	1.92	2.43

13	12.27	2	2	2	2-HIT, 1-TN	1.19	2.09

19	17.54	2	2	2	2-HIT, 1-TN	1.57	1.89

21	17.54	2	2	2	2-HIT, 1-TN	1.21	1.71

22	16.03	2	2	2	2-HIT, 1-TN	1.51	2.04

27	17.54	2	2	2	2-HIT, 1-TN	1.44	1.71

8	10.01	1	1	2	1-MISS	2.19	1.89

23	16.03	1	1	2	1-MISS	3.00	1.75

25	16.78	1	1	2	1-MISS	1.70	1.29

12	11.52	1	2	2	1-FALSE POSITIVE	1.51	1.92

18	14.53	1	2	2	1-FALSE POSITIVE	1.70	2.11

7	7.00	2	1	1	2-FALSE POSITIVE	2.12	1.90

17	13.02	2	1	1	2-FALSE POSITIVE	2.01	1.82

6	7.00	1	2	1	1-PSEUDO HIT	1.48	2.77

14	12.27	2	1	2	2-PSEUDO HIT	2.14	1.91

24	16.78	2	1	2	2-PSEUDO HIT	1.36	1.19

1	7.00	2	0	1	GUESS	1.70	1.72

15	12.27	2	0	1	GUESS	1.21	1.25

16	13.02	1	0	1	GUESS	1.60	1.58

28	17.54	1	0	1	GUESS	1.46	1.55

29	17.16	2	0	1	GUESS	1.79	1.79

For 66 year-old female f66z067.

### Ensemble Averaging

Our classification method based on anomaly detection used the information content in the APCoP signal as its primary measure, with the measurement at any particular instant based on only a small window of the data. If we clustered data runs based on this scheme, we could ensemble average across like-classified runs to compute a point-by-point ensemble average of the time series. In this methodology, we could then look for observable patterns and behaviours in the ensemble average, using those patterns to infer behavior of the postural control system.

**Figure 14:**
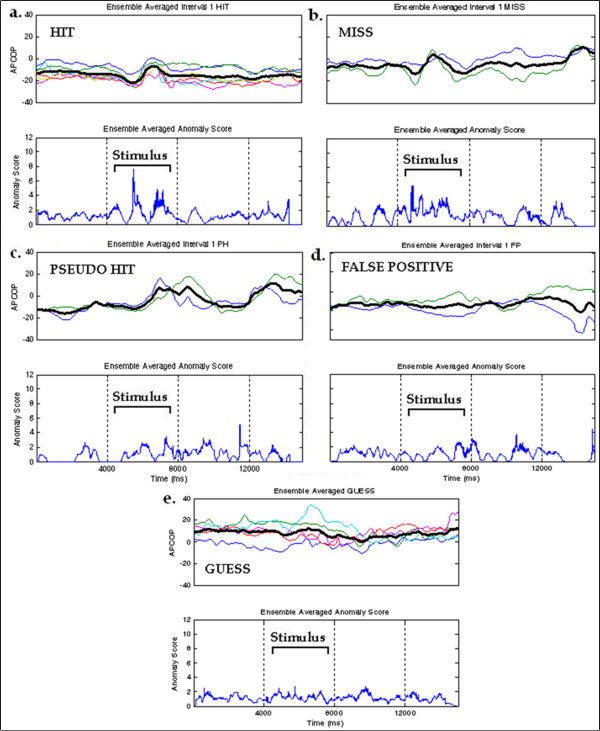
**Ensemble Averaged Profiles of APCoP and Anomalies**. (a) Interval 1 HIT, (b) Interval 1 MISS, (c) Interval 1PSEUDO HIT, (d) Interval 1 FALSE POSITIVE, (e) GUESS for the subject whose data is in Table 3.

Ensemble averages were constructed via point-by-point time averages of the data time-series acquired from 29 trials in a 16 mm run for the subject of Table 2. This technique amplified the anomalous behavior of the APCoP response and showed significant anomalies present in the expected interval for each category. The ensemble averaged profiles of 1-HIT, 1-MISS, 1-PH, 1-FP, and GUESS cases respectively are shown in Figure 14. Visually, it appeared this refined classification was now able to appropriately group the data runs, such that the ensemble average was a good representation of the group. We found similar results for the Interval 2 profiles.

### Group Responses

The classification procedure described above was applied to classification results summarized in Table 3 for all subjects. One additional subject from that test group was analyzed, but these anomaly scores were extreme outliers, and the original PEST experiment failed to find a threshold for this subject. These separate indicators warranted removal of this subject's data from the group analysis.

**Table 3:** Summary classification results for the 13 subjects.

Subject	Hit	Miss	Pseudo-hit	False Pos	Guess - Correct	Guess - Incorrect	Trials
f50z031	13	10	1	3	1	2	30

f51z065	17	7	3	2	1	0	30

f51z160	22	1	2	1	2	2	30

f53z077	9	2	4	4	2	2	23

f57z088	17	2	4	3	4	0	30

f58z097	19	3	3	2	3	0	30

f60z025	14	2	1	2	1	0	20

f62z021	25	4	0	1	0	0	30

f66z067	14	3	3	4	2	3	29

f67z125	20	6	1	0	1	2	30

f67z161	16	11	1	1	1	0	30

m50z028	16	7	2	2	2	1	30

m64z011	14	7	3	3	3	0	30

**Totals**	**216**	**65**	**28**	**28**	**23**	**12**	**372**

Subject codes listed in the first column provide the following information: m/f - Gender, next two digits - age, z - testing group, last two digits - subject number.

Under the assumption that the marginal data provided a useful representation of population behavior, we estimated the conditional probabilities associated with the various classifications. For example, the proportion of *correct *answers when the signal was *detectable *(based on APCoP anomaly score) was approximately 0.77, with 95% confidence interval

(5) .71≤P(C|D)≤.82

using a binomial model. Similar, the probability of *correct *given that APCoP data was *misleading *was 0.5, with 95% confidence interval

(6) .36≤P(C|M)≤.64

We note that, if the subjects were using only the APCoP data to decide on interval, then the misleading signals should have generated a much lower rate of correct responses. The ability to (sometimes) disregard the APCoP indicator and answer correctly when that signal was misleading might contribute in explaining the 25% error rate observed when the signal was detectable.

As the final marginal probability, we observed that the proportion of *correct *responses when the signal was classified as *guess *was 0.66, with confidence interval

(7) .48≤P(C|G)≤.81

If these runs were a pure guess (with no information available to the subject), we would have expected approximately 50% of the response to be correct. Although the 95% confidence interval did preclude this pure guess possibility, the one-tailed p-value was p = 0.045, indicating that there was statistical evidence that the rate was higher than 50%. The result could be viewed as consistent with the other proportions in that it provided evidence that the body was using additional indicators beyond the CoP signal to assess for movement of the platform. We note that this implication held only for the group statistics, and not for an individual, as we observe that much of the deviation from pure guess could be associated to the performance of three subjects (m64z011, f57z088, f58z097), with the rest of the group having approximately equal number of correct and incorrect responses for the *guess *categorization.

## Discussion

A primary contribution of this paper is the development of two new outcome categorizations for a 2AFC trial. This analysis yielded five categories and a guess. The novel classification scheme helped us understand psychophysical postural detection strategies when a subject was presented with the small anterior perturbations at the borderline of movement perception. To our knowledge, all previous work had simply described the outcome as either "correct" or "incorrect," depending on whether the subject correctly identified the presented stimulus. Also, traditional signal detection could only yield four categories, HIT, MISS, FALSE ALARM, and CORRECT REJECTION. Our innovative analysis introduced a new category called 'PSEUDO HIT', and identified a way to distinguish a GUESS where traditional signal detection theory cannot.

As our secondary contribution, we present a methodology that classifies psychophysical detection strategies based on the information content of the biomechanical data. By assessing the anomaly of the biomechanical measurement, we could determine whether there was sufficient information available such that the signal was detectable. Our key innovation is that, rather than using the full time-series of information to discover data patterns, the analysis technique should respect the psychology of the experiment. As consequence, we were able to classify the biomechanical information in our experiments as either indicating that there was a detectable signal or that there was insufficient information that the signal was actually misleading with respect to the decision task at hand.

We applied a modified time-series-bitmap based approach to analyze human postural control. We remark now that analysis via our "anomaly score" metric should be viewed as vastly different from standard linear filtering techniques, using a non-linear discretization of the data to maximize the information content while using a very small alphabet of states. The discretization enabled us to transform the data in a second non-linear process, which identified anomalies by comparison of probability distributions, which in turn can be related to Markov models and information theoretic approaches such as cross-entropy or Kullback-Leibler divergence [21]. Although not presented in this paper, we also examined a vast array of standard filters applied to this problem. We chose to pursue the non-linear transformation described in this paper because we were unable to find a standard linear filtering (to include examination of signal derivatives) that allowed us to differentiate within the expected biomechanical detection response behaviours. The techniques of this paper were closely related to the work of Lin, Keogh, Wei, and their collaborators [11,20,22], with slight modifications in method that significantly affected their applicability to the 2AFC environment: (a) by considering only a small (moving) lead-lag window, our method identified "anomaly" as those time periods where the signal was changing rapidly in character, as opposed to where the signal was being differentiated from the full time series, (b) we used knowledge of the experiment to average over the specific test interval in the same way that the subject was asked to differentiate. This analysis allowed us to classify runs based on the psychological and biomechanical implications of the experiment. The resultant ensemble averages then served as *motifs *to characterize specific human behaviours of the experiment.

The implication of these departures from the standard TSB based methods is significant. As described [20], motifs can be identified in data sets, but the analysis considers the whole time series. If data runs were grouped in this way, the only conclusion to draw was that "this group of time series signals was similar," without any valid way to associate them to a specific psychophysical or biomechanical situation reflective of the experiment. A similar weakness would apply to any attempt to classify the signals via pattern matching and identification. Consequently, we claim as a very important conclusion that the motifs identified by our method are descriptive of important psychophysical aspects of the experiment.

As additional remark on these methods, we note that the data reduction technique relied on a discretization based on an assumption that the data was approximately normally distributed. For the APCoP signal, we found this to be reasonable. For other physiological data collected in this experiment (such as EMG data), other discretization schemes could be required. However, regardless of the method used to partition the space and assign symbols to the time-series data, the rest of the method (comparison using frequency tables) can still be directly applied.

Another major contribution was our pairing a biomechanical indicator with a psychophysical indicator in a 2AFC environment, providing a unique characterization that (to our knowledge) has not been previously described. Our categorization of trials into HITs, MISSes, PSEUDO HITs, FALSE POSITIVEs, TRUE NEGATIVEs, and GUESSes provided a detailed view of the experimental results that would not be possible if we considered only the subject's stated response. We would imagine that in other 2AFC experiments, modern data collection schemes (e.g., iris tracking in a visual perception experiment) might easily provide the necessary biomechanical data to allow similar classification. To our knowledge, this refined characterization scheme for the 2AFC experiments represents a major contribution in the psychophysical literature. Additionally, we believe our techniques have broader applicability in other psychophysical applications of threshold detection.

### Biomechanical Event

A primary conclusion was that the APCoP signal could be used to assess the effect of platform movement on body motion under the low level perturbations of the experiment, though not with complete accuracy (81.4% correct). For those situations where there was a detectable signal, even then many subjects answered correctly on only about 3 out of 4 trials, indicating that (a) the subject could not correctly interpret the APCoP information or (b) the subject used other additional information in making the perceptive decision. However, we do note that most psychophysical testing defines threshold at 75%, thus the 81% was above that value. That other information was used appears to be supported by higher expect rates of *correct *responses for both the *misleading *and *guess *APCoP signals. We intend to pursue richer data models to support an analysis of collected EMG data to better capture this aspect.

One source of conceptual error in our model was the assumption that the APCoP signal was fully describing the postural response to platform perturbations. Although valid at a superficial level, we noted that the APCoP signal did reflect a postural response, regardless of the initiating signal, to include conscious movement and subconscious reactions of the subject. As anecdotally observed, a subject who was immediately convinced that they were sensing platform movement in interval one would "relax" in interval two, where normally the relaxation took place in the "decide" interval of the experiment. As such, the body motion in interval 2 was "anomalous," perhaps at levels larger than might have been caused by an interval 1 stimulus. The subject's correct response to interval 1 move would be recorded as a PSEUDO-HIT, but that classification was actually the result of a "correct detection" in interval 1.

The *GUESS *categorization can be viewed as somewhat of a misnomer, with a better description being that those trials were *ambiguous *with regard to APCoP information. It was likely that other information was used to provide the subject with greater accuracy than a pure guess. With the small size of the data sets, however, it was not clear whether this group statistic reflected that *some *subjects were using additional information while most were not. We have evaluated other choices for the guess threshold 0.05 ≤ a_t _≤ 0.25, and within this ranges the results were reasonably consistent in that there was a borderline distinction that might have been associated with just a few individuals. Choosing a smaller threshold significant reduced the sample size, while choosing a larger value began to bring in more trials where the anomaly difference provided a clear indication of the stimulus. We are now exploring additional statistical techniques to attempt to better resolve the guess-like behavior.

In the future, we wish to apply this classification to subjects of a wide age range, with and without diabetes. We expect that the refined analysis based on an ensemble average taken across the appropriate classifications developed in this paper will support more rapid discovery of postural control behaviours. The ensemble averaging employed to date has focused on averaging across a single individual, resulting in a motif description of that category. Our next step is to explore those motifs across a range of subjects to identify common characteristic on the group. For example, we want to characterize the features of a HIT, where those features are common to the ensemble average of all the subjects in a group. Also, our immediate focus is on how EMG data might be used to improve the accuracy of the classifier.

Based on this work, a physiological or biomechanical threshold detection experiment could be designed to see whether a change in the APCoP (or some other variable) corresponds to a presentation of a stimulus, independent of a psychological response or choice. The classification of "detectable" is currently only a description of whether motion is detectable in the CoP signal. As the further analysis might reveal how the body uses other biomechanical indicators to determine a perturbation, we intend to incorporate those features into the classification methodology. This new threshold detection technique could help in designing threshold iteration schemes in a way that could automatically account for GUESSes and PSEUDO HITs without requiring a psychophysical response (e.g., a button press) from the subject. Such an automated threshold detection mechanism could provide a new tool not only to study human balance mechanisms, but also to investigate true detection thresholds for other sensory modalities.

## Competing interests

Initial data collection was supported by a VA Senior Rehabilitation Research Career Scientist Award to C. J. Robinson, and VA Rehabilitation R&D grants 91-355AP, E2143R, and E01-2097R. Later data collection and analysis were supported by NIH R01AG026553 and by a Coulter Foundation endowment to Clarkson University. J.D. Skufca is also supported by NSF Grant DMS-0404778.

## Authors' contributions

VVB carried out anomaly detection analysis, developed a new classification scheme, and drafted the manuscript. JDS proposed the alternative probabilistic model and assisted in choosing a threshold value to define guesses. RBP assisted in development of the classification scheme and threshold analysis. CMS contributed in designing the data collection protocol and data acquisition. As the principal investigator, CJR oversaw the experimental design and set up, and helped to draft and edit the manuscript. All authors have read and approved the final manuscript.
